# Acute Sedentary Behaviour and Markers of Cardiometabolic Risk: A Systematic Review of Intervention Studies

**DOI:** 10.1155/2012/712435

**Published:** 2012-06-17

**Authors:** Travis J. Saunders, Richard Larouche, Rachel C. Colley, Mark S. Tremblay

**Affiliations:** ^1^Healthy Active Living and Obesity Research Group, Children's Hospital of Eastern Ontario Research Institute, Ottawa, ON, Canada K1H 8L1; ^2^School of Human Kinetics, University of Ottawa, Ottawa, ON, Canada K1N 6N5; ^3^Department of Pediatrics, University of Ottawa, Ottawa, ON, Canada K1N 6N5

## Abstract

North Americans spend half their waking hours engaging in sedentary behaviour. Although several recent interventions suggest that short bouts of uninterrupted sedentary behaviour may result in acute increases in cardiometabolic risk, this literature has not been reviewed systematically. This study performed a systematic review of the impact of uninterrupted sedentary behaviour lasting ≤7 days on markers of cardiometabolic risk (insulin sensitivity, glucose tolerance, and fasting insulin, glucose, and lipid levels) in humans. Interventions were identified through systematic searches of Medline and Embase and screened by 2 independent reviewers. A total of 25 interventions were identified that examined the impact of imposed sedentary behaviour on biomarkers of interest. The majority of these studies focused on healthy young men, with very little identified research on females or other age groups. We found consistent, moderate quality evidence that uninterrupted sedentary behaviour ≤7 days results in moderate and deleterious changes in insulin sensitivity, glucose tolerance, and plasma triglyceride levels. In contrast, there is inconsistent, very low-quality evidence linking uninterrupted sedentary behaviour with changes in insulin, glucose, and HDL- and LDL-cholesterol levels. These findings suggest that uninterrupted bouts of sedentary behaviour should be avoided in order to prevent or attenuate transient increases in metabolic risk.

## 1. Introduction

High levels of chronic sedentary behaviour are associated with increased risk of obesity [1, 2], diabetes [1, 3], cardiovascular disease [4–6], some cancers [7], and even mortality [3–5]. For example, it has been reported that sedentary behaviour is prospectively associated with increased risk of all-cause and cardiovascular disease mortality, and that these associations remain significant after control for physical activity, diet, and smoking [4]. These findings suggest that sedentary behaviour should not be viewed as simply the lack of physical activity but may instead represent an independent and distinct risk factor for chronic disease.

In addition to the health impact of chronic sedentary behaviour, recent evidence suggests that increases in metabolic risk may be apparent following bouts of uninterrupted sedentary behaviour lasting just a few days in length [8–11]. Stephens et al. reported that a single day of uninterrupted sitting resulted in a 39% reduction in whole body insulin action in healthy adults [11]. Similarly, Hamburg et al. observed that 5 days of continuous bed rest produced deleterious changes in cholesterol, triglyceride, glucose, and insulin levels and reduced insulin sensitivity [10]. These findings are supported by work in animal models, which suggest that just 6–24 hours of sedentary behaviour results in significant reductions in lipoprotein lipase activity [12] and insulin sensitivity [13, 14] in skeletal muscle.

Although several narrative reviews have discussed the acute changes in metabolic risk following short-term exposure to uninterrupted sedentary behaviour [8, 9, 15, 16], the published literature in this area has yet to be examined systematically. Therefore, we conducted a systematic review examining the impact of uninterrupted sedentary behaviour lasting ≤7 days (operationally defined as an “acute” bout) on insulin sensitivity, glucose tolerance, and lipid, glucose, and insulin levels in humans.

## 2. Methods

### 2.1. Study Criteria

To be included in this paper, a study had to examine at least one of the following risk markers in humans: insulin sensitivity, glucose tolerance, or fasting insulin, glucose, or lipid levels. Uninterrupted sedentary behaviour had to be imposed by the researchers for a period lasting 7 days or less. Studies examining longer (e.g., chronic) bouts of sedentary behaviour were excluded as it was felt that it would be inappropriate to consider the impacts of both acute and chronic sedentary behaviour in a single systematic review, given the large amount of heterogeneity that this would introduce into the methodologies of included studies. Only intervention studies (both randomized and nonrandomized) that imposed on participants a controlled bout of sedentary behaviour were included in this paper. 

For the purposes of this paper, sedentary behaviour was defined as a distinct class of waking behaviours characterized by little physical movement and low-energy expenditure (≤1.5 METs), as well as a sitting or reclining posture [9]. Eligible forms of sedentary behaviour included sitting, bed rest (head-up, horizontal, and head-down), and casting (e.g., having one or more legs immobilized in a cast). There were no restrictions placed on the age or sex of participants. Only articles published in English or French were included in the present review, and no limits were placed on the date of publication. The review methodology was prospectively registered in PROSPERO (Registration number: CRD42011001431). 

### 2.2. Search Strategy

Literature searches were performed using Ovid Medline and Ovid Embase in March of 2012. The search strategy was created with the help of a research librarian and run by TJS (see Algorithm 1). Potentially relevant articles were also identified by 6 key informants and through the authors' personal reference libraries. Articles were extracted as text files from the Ovid interface and imported into Reference Manager (Thompson Reuters, San Fransisco, CA, USA). Duplicate articles were first removed using the Ovid interface, and any remaining duplicates were removed manually. Once imported into Reference Manager, conference abstracts were also removed from the database. 

Titles and abstracts of articles identified through the search were reviewed by two authors (T. J. Saunders and R. Larouche) using Reference Manager. Any articles identified as being potentially relevant by either reviewer were obtained for further screening. The full text of these articles was then reviewed independently by TJS and RL to determine whether the article met the *a priori *review inclusion criteria. All decisions at this stage were made by consensus and any discrepancies between the two reviewers were resolved through discussion. In this paper consensus was reached for all included articles. 

### 2.3. Data Extraction and Analysis

Data was extracted by T. J. Saunders and verified by R. Larouche. Information was extracted regarding study design (year, methodology, country, number of participants, duration of sedentary behaviour, age), modality of sedentary behaviour, risk factors examined, and main findings. Reviewers were not blinded to the authors or journals when extracting data. The primary summary measure was the mean difference in each outcome measure (or mean change in nonrandomized interventions) following exposure to acute sedentary behaviour. Where possible, effect sizes were calculated using Cohen's *d*. We defined an effect size of ≤0.20 as small, an effect of 0.21–0.80 as moderate, and effects ≥0.81 as large. For the purposes of this paper, positive effect sizes represent increased cardiometabolic risk (e.g., increased fasting triglyceride levels), while negative effect sizes represent reduced risk. 

Following data extraction it became clear that the interventions included in the present paper were very heterogeneous in terms of the length of exposure, the type of sedentary behaviour that was examined, and even the measurement of individual risk factors (e.g., insulin sensitivity was assessed using HOMA, QUICKI, whole body insulin action, oral glucose tolerance tests, and hyperinsulinemic clamps). Thus, we believe that meta-analyses or pooling of data across studies would be inappropriate and have therefore performed a qualitative synthesis of the evidence instead. 

Forest plots were created using Review Manager 5.1 (The Nordic Cochrane Centre, The Cochrane Collaboration) to display the relationship between sedentary behaviour and each outcome of interest. Studies assessing glucose tolerance and insulin sensitivity employed a wide range of methodologies and units of measurement, and plots for these outcomes are therefore presented as percent mean difference, while all other outcomes are presented as mean difference with 95% confidence interval. Studies which did not provide raw data were not included in forest plots. 

### 2.4. Quality of Evidence

The risk of bias and strength of evidence from individual studies was assessed using the Downs and Black Checklist [17]. This 27 point checklist assesses the strength of reporting, external validity, internal validity, and power. As some questions are worth more than one point, the maximum score that a study can receive is 32. 

The quality of evidence for each outcome was assessed as high, moderate, low, or very low using the GRADE approach [18]. In this approach, randomized trials begin as high-quality evidence and observational studies begin as low-quality evidence. For the purposes of this paper, nonrandomized interventions were considered as observational studies. Following the initial rating based on study design, the quality of evidence was then rated up or down for apparent risk of bias, imprecision, inconsistency, indirectness, or suspicion of publication bias. Risk of bias was assessed using Review Manager Version 5.1 (The Nordic Cochrane Centre, The Cochrane Collaboration), and GRADE was assessed using GRADEpro Version 3.6 (GRADE Working Group). 

## 3. Results

### 3.1. Description of Studies

**Figure 1:**
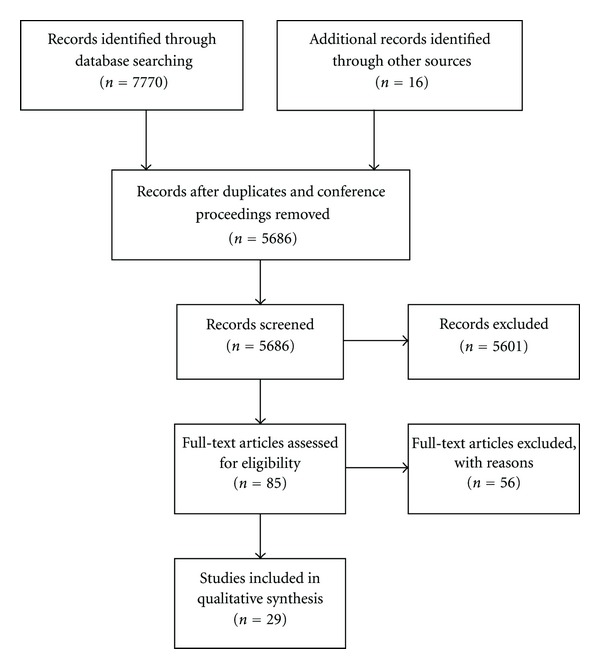
Flow of articles through the search process.

After deduplication and the removal of conference abstracts the search strategy retrieved 5,670 articles for initial screening (Figure 1). To this, 16 additional articles that were identified through key informants were added, bringing the total number of potential articles to 5,686. Initial screening of titles and abstracts identified 85 articles that received a detailed assessment of the full text article. Reasons for excluding studies included an ineligible exposure (e.g., the bout of sedentary behaviour exceeded 7 days, or simply investigated the impact of reducing structured physical activity in active individuals, without actually imposing sedentary behaviour) (*n* = 26), the article being written in a language other than English or French (*n* = 12), ineligible outcome (*n* = 7), the article being a review or commentary (*n* = 10), and “other” (*n* = 2). Some articles were excluded for multiple reasons. 

A total of 29 articles reporting data from 25 independent interventions met all inclusion criteria and are presented in the current review. Nineteen of the identified interventions were nonrandomized trials, 4 were randomized crossover studies (e.g., participants served as their own controls), and 2 were randomized controlled trials. The studies included a total of 368 participants (309 males and 59 females), who were recruited from 12 countries across North America (USA), Europe (Denmark, France, Bulgaria, Russia, Greece, Sweden, Poland, and Slovakia, Norway), Asia (Japan), and Oceania (Australia). Participants ranged from 18 to 72 years of age, although the average age of participants was under 35 years for all but 3 studies, and under 30 for all but 7 studies. Sixteen studies employed head-up or horizontal bed rest, 5 employed head-down bed rest, 4 employed sitting, and one employed casting (one employed both sitting and head-down bed rest). The smallest studies had 5 participants [19, 20] and the largest had 38 [21]. The mean number of participants per study was 15.1 ± 10.1, and the median was 10. 

Three studies examined the impact of 2, 4, and 5 hours of uninterrupted sedentary behaviour on biomarkers of interest, respectively; all other studies examined the impact of 1 day or more. Six studies examined the impact of 1 day of sedentary behaviour, 4 examined 2 days, 7 examined 3 days, 2 examined 4 days, 6 examined 5 days, 2 examined 6 days, and 8 examined the impact of 7 days of sedentary behaviour (6 studies collected data at multiple time-points). Characteristics of individual studies are presented in Table 1. 

### 3.2. Fasting Insulin

**Figure 2:**
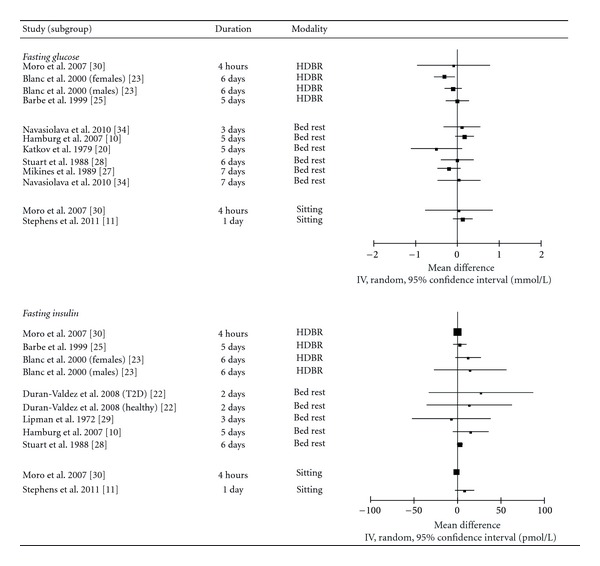
Forest plot of mean differences of fasting glucose and insulin values between sedentary behaviour and control conditions (sedentary behaviour-control).

Two randomized crossover studies (*n* = 22) [11, 22] and 12 nonrandomized intervention studies (*n* = 185) [10, 21, 23–32] examined the impact of sedentary behaviour on fasting insulin levels (Figure 2). Neither randomized crossover study observed significant changes in insulin levels as a result of uninterrupted sedentary behaviour, despite having effect sizes in the moderate range. Stephens et al. [11] reported that fasting insulin levels were 47.6 ± 11.6 pmol/L following one full day (17 hours) of uninterrupted sitting, compared to 39.3 ± 16.3 pmol/L following a day that included as little sitting as possible (~6 hours of sitting spread throughout the day) in a group of 12 healthy young adults. Duran-Valdez et al. [22] also observed nonsignificant increases following 2 days of strict bed rest in both healthy participants (71.4 ± 42.6 versus 84.1 ± 36.4 pmol/L) and those with type 2 diabetes (79.2 ± 50.3 versus 106.3 ± 46.6 pmol/L). 

Three nonrandomized interventions reported significant increases in insulin levels ranging from 26 to 47% following uninterrupted sedentary behaviour [10, 23, 28] while 8 reported no change [21, 24–27, 29, 30]. The interventions that observed a change in insulin levels tended to impose uninterrupted sedentary behaviour for a longer period of time than those that found no change (6.0 ± 1.0 days versus 3.9 ± 2.6 days), although the mean number of participants (14.0 ± 7.0 versus 16.4 ± 13.3) and the quality of the studies did not appear to differ across the interventions. Effect sizes for these nonrandomized interventions ranged from −0.16 to 0.95, with all but one study reporting effect sizes in the small and moderate ranges. 

Given the aforementioned evidence from both randomized and nonrandomized interventions, we conclude that an acute bout of uninterrupted sedentary behaviour may result in a small-to-moderate increase in fasting insulin levels. However, the inconsistency of this effect and the lack of a statistical significance in randomized interventions leads us to conclude that the quality of this evidence is very low (Table 2). 

### 3.3. Fasting Glucose

Two randomized crossover studies (*n* = 22) [11, 22], one randomized controlled trial (*n* = 20) [33], and 14 nonrandomized intervention studies (*n* = 149) [10, 20, 23–30, 34–36] examined the impact of uninterrupted sedentary behaviour on fasting glucose levels. The one randomized controlled trial reported that, in comparison to ambulatory controls, plasma glucose levels were elevated by 34% following 7 days of uninterrupted bed rest [33]. The effect size in this randomized controlled trial was greater than 1, signifying a large effect. In contrast, neither randomized crossover study reported any change in fasting glucose levels following uninterrupted sedentary behaviour [11, 22]. One of these randomized crossover studies reported a moderate effect size of 0.43, [11], while the other reported small effect sizes of 0.07 and 0.14 in healthy participants and those with type 2 diabetes, respectively [22]. 

One nonrandomized intervention observed a significant increase in glucose levels of moderate size [10], one reported a significant reduction of moderate size [36], and one intervention observed moderate and large reductions in males and females, respectively, although this change was only significant in females [23]. The 11 other intervention studies did not observe any significant change in fasting glucose levels following uninterrupted sedentary behaviour [20, 24–30, 32, 34, 35]. The effect sizes among these 11 studies ranged from −1.21 to 0.47. 

Given the evidence provided by 17 separate intervention studies, we conclude that an acute bout of uninterrupted sedentary behaviour may result in a small-to-moderate increase in fasting glucose levels. However, the high level of inconsistency from both randomized and nonrandomized interventions leads us to conclude that this evidence is of very low quality. 

### 3.4. Fasting Triglycerides

**Figure 3:**
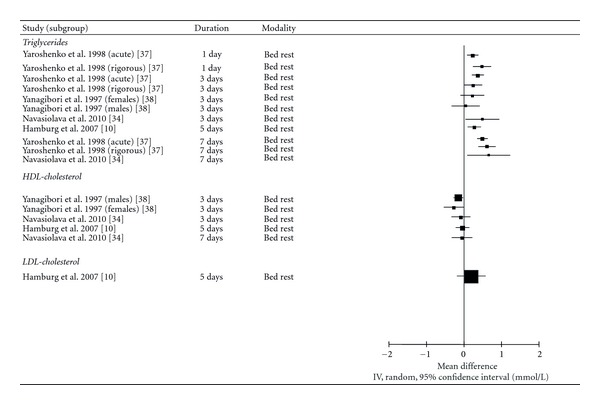
Forest plot of mean differences of fasting lipid levels between sedentary behaviour and control conditions (sedentary behaviour-control).

One randomized controlled trial (*n* = 30) [37] and 3 nonrandomized interventions (*n* = 51) [10, 34, 38] assessed the impact of uninterrupted sedentary behaviour on fasting triglyceride levels (Figure 3). The randomized controlled trial [37] exposed 20 men to one week of bed rest and assessed triglyceride levels on days 1, 3, and 7. The 20 men in the experimental group were further split into two groups of 10—those who knew when their bed rest would begin (*acute* bed rest), and those who were not told when it would begin (*rigorous* bed rest). In comparison to the control group, triglyceride levels were significantly elevated by 30.2% in the acute group after one day, although no change was observed in the rigorous group. Following 3 days of bed rest, triglyceride levels were elevated by 15.2% and 23.6% in the acute and rigorous bed rest groups, respectively. At the completion of 1 week of bed rest, triglyceride levels remained elevated by 36.8% and 31.9% in the acute and rigorous bed rest groups in comparison to the control group. The effect size for sedentary behaviour in this intervention was above 1 for both intervention groups on days 1, 3, and 7, indicating a large effect. 

The three nonrandomized interventions also found that acute sedentary behaviour resulted in significant increases in triglyceride levels [10, 34, 38]. Hamburg et al. [10] reported that triglyceride levels were elevated by 34.8% following 5 days of bed rest in 20 healthy men and women. Navasiolava et al. [34] observed that although no change in triglyceride levels was observed following 3 days of acute sedentary behaviour in a group of 8 male participants, triglyceride levels were 58.9% higher than baseline on day 7. Finally, Yanagibori et al. [38] found that triglyceride levels were elevated by 38.1% following 3 days of bed rest in men, but not women. With the exception of male participants in one study [38], the effect sizes reported in these nonrandomized interventions were all moderate to large.

Given the large and relatively consistent changes in triglyceride levels reported by both a randomized controlled trial and nonrandomized interventions, we conclude that acute bouts of uninterrupted sedentary behaviour result in a moderate-to-large increase in circulating triglyceride levels and that the available evidence is of moderate quality. 

### 3.5. Fasting HDL-Cholesterol

Three nonrandomized interventions (*n* = 51) [10, 34, 38] reported on the effect of uninterrupted sedentary behaviour ranging from 3 to 7 days on HDL-cholesterol levels. Two interventions reported nonsignificant reductions in HDL-cholesterol levels following sedentary behaviour [10, 34] while one study [38] reported significant reductions of 11.5% and 19.3% in men and women, respectively, following 3 days of bed rest. The effect sizes in these studies ranged from 0.09 to 0.84, suggesting that acute bouts of uninterrupted sedentary behaviour may result in moderate reductions in HDL-cholesterol levels. However, given the inconsistency of these findings and the lack of data from randomized interventions, we conclude that the available evidence is of very low quality. 

### 3.6. LDL-Cholesterol

Two nonrandomized interventions (*n* = 28) [10, 34] examined the relationship between uninterrupted sedentary behaviour and changes in LDL-cholesterol levels following 3 [34], 5 [10], and 7 [34] days of sedentary behaviour. Although the studies reported moderate-sized increases in LDL-cholesterol levels at all time points, none of these increases were statistically significant. Thus, while the available evidence suggests that an acute bout of uninterrupted sedentary behaviour may result in a moderate increase in LDL-cholesterol levels, the quality of this evidence is very low. 

### 3.7. Insulin Sensitivity

**Figure 4:**
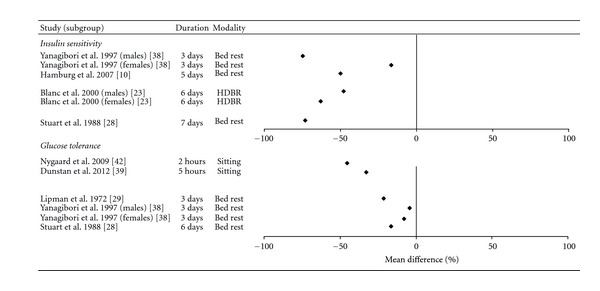
Forest plot of percent mean differences of insulin sensitivity and glucose tolerance between sedentary behaviour and control conditions (sedentary behaviour-control).

Three randomized crossover studies (*n* = 41) [11, 22, 39] and 10 nonrandomized interventions (*n* = 120) [10, 19, 23, 24, 28, 29, 32, 38, 40, 41] examined the relationship between acute bouts of uninterrupted sedentary behaviour and measures of insulin sensitivity in healthy adults (Figure 4). The measures employed included HOMA [10, 32, 41], QUICKI [22], insulin-stimulated glucose uptake [11], insulin sensitivity index [10], insulin area under-the-curve (AUC) during oral glucose tolerance tests or standardized meals [23, 24, 28, 38, 39, 41], and hyperinsulinemic euglycemic clamps [19, 28, 40]. The crossover studies measured insulin sensitivity during 5 hours of sedentary behaviour [39], as well as before and after 1 [11, 22] and 2 [22] days of sedentary behaviour. The nonrandomized interventions assessed insulin sensitivity before and after 3 [24, 29, 38], 5 [10], 6 [23], and 7 [19, 28, 40, 41] days of sedentary behaviour. 

Two of the three randomized crossover studies [11, 39] reported that uninterrupted sedentary behaviour had a deleterious effect on insulin sensitivity. Stephens et al. reported that insulin-stimulated glucose uptake was 39% lower following a day of acute sitting in a group of 12 healthy adults, in comparison to a day that minimized sitting [11]. Similarly, Dunstan et al. reported that the insulin AUC following a standardized meal was increased by 30% following 5 hours of uninterrupted sitting in a group of 19 overweight adults, in comparison to 5 hours of sitting which was broken up with periodic light-intensity walk breaks [39]. The third crossover study [22] reported that 1 day of sedentary behaviour resulted in a nonsignificant reduction in QUICKI scores in 5 healthy adults and a nonsignificant increase in 5 adults with type 2 diabetes. A significant reduction in insulin sensitivity was observed following two days of bed rest in participants with type 2 diabetes, but not in healthy adults. The significant reduction in participants with type 2 diabetes following 2 days of bed rest was of moderate size, while the nonsignificant reduction in healthy participants was small. 

Eight of the 10 nonrandomized trials reported significant reductions in insulin sensitivity ranging from 12.5% to 100% following uninterrupted sedentary behaviour. For example, Hamburg et al. [10] reported that HOMA insulin sensitivity was reduced by 50% following 5 days of bed rest in 20 healthy adults, while the insulin sensitivity index was reduced by 12.5% in the same group of subjects. Similarly, Yanagibori et al. [38] report that insulin AUC during an oral glucose tolerance test was increased by 16.6% in 10 men and 74.9% in 7 women following 3 days of bed rest. The effect sizes from these nonrandomized interventions ranged from 0.34 to 3.3. 

Although the majority of studies (9/12) examining insulin sensitivity had no control group, the effect sizes of the sedentary behaviour interventions were consistently moderate to large. The results were also consistent, with 10 of 12 published studies reporting a reduction in insulin sensitivity in at least one subgroup of participants. Thus, we conclude that acute bouts of uninterrupted sedentary behaviour are likely to result in a moderate-to-large reduction in insulin sensitivity and that the available evidence is of moderate quality. 

### 3.8. Glucose Tolerance Tests

Two randomized crossover studies (*n* = 32) [39, 42] examined the impact of 2 [42] and 5 hours [39] of prolonged sitting on glucose AUC in response to a standard meal, while seven nonrandomized interventions (*n* = 87) [23, 24, 27–29, 38, 41] examined the impact of uninterrupted sedentary behaviour lasting 3 [24, 38], 6 [23], and 7 [27, 28, 41] days on measures of glucose tolerance. 

Both of the randomized crossover studies reported that uninterrupted sitting resulted in significant increases in glucose AUC in response to a standardized meal. Nygaard et al. reported that 2 hours of sitting resulted in a 45% increase in the glucose response to a standard meal in a group of 13 elderly women, in comparison to a combination of sitting and walking at a self-selected “very light” intensity [42]. Similarly, Dunstan et al. reported that the glucose AUC following a test meal was 33% higher following 5 hours of prolonged sitting, in comparison to 5 hours of sitting which was broken up with periodic light-intensity walk breaks [39]. 

Five of seven nonrandomized studies reported significant reductions in glucose tolerance in at least some participants, ranging from 7.8 to 30%. For example, Smorawiński et al. [24] reported that glucose AUC during oral glucose tolerance tests was 30% higher following three days of bed rest in inactive young men, although there were no change in endurance- or strength-trained athletes. Yanagibori et al. [38] observed significant 7.8% reductions in oral glucose tolerance in women, but not men, following 3 days of bed rest. The effect sizes in these studies ranged from −0.03 to 1.4 and were in the moderate or high range for all but one study. 

The available evidence suggests that acute bouts of uninterrupted sedentary behaviour may result in moderate-to-large reductions in oral glucose tolerance. Given the relatively consistent findings and the strong evidence from randomized crossover studies, we conclude that the evidence linking acute sedentary behaviour with reductions in glucose tolerance is of moderate quality. 

### 3.9. Quality Assessment

Downs and Black scores assessing the risk of bias for individual studies are presented in Table 1. The average score was 21.4 ± 2.3, out of a maximum of 32. The three randomized crossover studies had the highest quality (25.0 ± 1.7), followed by the randomized controlled trials (22.5 ± 2.1) and the nonrandomized interventions (20.8 ± 1.9). The overall quality of evidence related to each outcome is presented in Table 2. 

## 4. Discussion

Based on our systematic review of data from 25 independent interventions, we found moderate quality evidence suggesting that acute bouts of uninterrupted sedentary behaviour lasting 2 hours to 7 days result in rapid and deleterious changes in triglyceride levels, insulin sensitivity, and glucose tolerance. We also found very low-quality evidence that it results in changes in fasting glucose, fasting insulin, and HDL- or LDL-cholesterol. 

The findings of the current paper have important public health implications. Recent estimates suggest that on average North American adults and children spend 7–10 hours per day—more than half their waking hours—engaging in sedentary behaviour [43–46]. This suggests that many individuals likely spend several consecutive hours sitting down on a regular basis, which is not dissimilar to the protocol employed by 3 randomized crossover studies in this paper that resulted in significant reductions in insulin sensitivity and glucose tolerance [11, 39, 42]. Individuals who perform long bouts of uninterrupted sedentary behaviour on a regular basis may therefore be exposing themselves to higher levels of circulating triglycerides, as well as reduced insulin sensitivity and glucose tolerance, which may help to explain the prospective associations between sedentary behaviour and chronic disease morbidity and mortality [3–5]. 

Research in animal models suggests mechanisms that may explain our observation of consistent changes in both insulin sensitivity and plasma triglyceride levels in response to uninterrupted sedentary behaviour. Bey and Hamilton reported that just 18 hours of hindlimb unloading results in near total cessation of lipoprotein lipase activity and roughly 75% reduction in triglyceride uptake in rat skeletal muscle [12]. Similarly, it is also well established that skeletal muscle denervation results in rapid changes in glucose transport protein expression and reductions in insulin sensitivity [13, 14]. These findings suggest that rapid and deleterious changes in skeletal muscle metabolic function may underlie the relationship between sedentary behaviour, triglyceride levels, and insulin sensitivity observed in the present review. 

### 4.1. Strengths and Limitations

The major strength of this paper is its rigorous systematic methodology. The search strategy was developed in consultation with a research librarian with expertise in search creation, and the screening process included two independent reviewers who came to consensus on all included studies. Strength of evidence was assessed using GRADE in order to increase the transparency of the grading process. Finally, the paper was prospectively registered with PROSPERO. 

The limitations of this paper relate primarily to the quality of evidence that is presently available. Of 25 independent interventions identified by this paper, only 6 employed a randomized design. Further, although fasting glucose, glucose tolerance, insulin, and insulin sensitivity have each been examined by 9 or more investigations, lipid levels have received little attention by comparison. Given the small number of studies and the low quality of evidence currently available for these outcomes, it is difficult to determine their relationship with sedentary behaviour with any certainty. 

There has also been a large amount of heterogeneity in the modality of sedentary behaviour (e.g., sitting versus bed rest) and in the way that outcome measures are calculated, which precluded the use of meta-analyses in the present paper. Only 5 studies identified by the current paper examined a modality of sedentary behaviour other than bed rest. The modality of sedentary behaviour which is most common in daily life is undoubtedly sitting, yet the acute impact of sitting has only been examined in four interventions. In contrast the metabolic impact of bed rest has received far more attention in the published literature [16], despite the fact that prolonged periods of bed rest are uncommon in day-to-day life. Given that it is unclear whether sitting and bed rest have a similar impact on markers of cardiometabolic risk, it is important that future studies focus on the impact of sitting to determine whether it has an impact which is similar to that of bed rest. 

The sample size of most interventions identified by this paper was quite small, and the vast majority of studies were performed in physically fit, healthy young adult males between the ages of 20 and 30. We only identified two interventions focused on individuals above the age of 50 [39, 42], or those with elevated body weight [22, 39] and we were not able to identify any interventions focusing on pediatric populations. Further, females made up just 16% of the participants in the identified interventions, which makes it unclear whether the relationships observed in the current paper will generalize to females of any age. 

It is also worth noting that, at present, it is difficult to differentiate the impact of sedentary behaviour *per se* from that of a positive energy balance. If energy intake is maintained at an individual's habitual level, it can be assumed that an imposed bout of sedentary behaviour is likely to result in positive energy balance. However, to our knowledge only one intervention [11] has attempted to separate the impact of an acute bout of sedentary behaviour from that of acute positive energy balance. Interestingly, Stephens et al. report that reducing energy intake to match energy expenditure during a bout of prolonged sedentary behaviour reduced the deleterious impact on insulin sensitivity by roughly 50% [11]. Further, no studies identified in the current paper reported adjusting results for baseline physical activity, fitness, or diet. Future work should investigate these issues further, in order to determine the relative contributions of sedentary behaviour and positive energy balance to changes in cardiometabolic risk factors. 

To date only three studies have examined the impact of uninterrupted sedentary behaviour lasting less than 1 day on markers of metabolic risk. Given that healthy individuals rarely spend 24 hours engaging in uninterrupted sedentary behaviour, it is important that future studies investigate whether shorter bouts of sedentary behaviour also have a measurable impact on metabolic health. Future work should also investigate the acute impact of sedentary behaviour on nontraditional markers of cardiometabolic risk including adipokines and markers of inflammation. Finally, none of the studies identified in the current paper examined whether these deleterious changes in risk markers persisted once participants returned to free living conditions. Thus, it is unclear whether the changes observed in the reviewed studies endure for several days following the cessation of sedentary behaviour, or whether they are rapidly resolved. Assessing the clinical significance of these changes will be difficult until their time-course has been more carefully examined. 

## 5. Conclusions

This study demonstrates that, at present, there is moderate quality evidence that acute bouts of uninterrupted sedentary behaviour result in significant and deleterious changes in insulin sensitivity, glucose tolerance, and plasma triglyceride levels. There is currently very low-quality evidence linking uninterrupted sedentary behaviour with changes in circulating insulin, glucose, and HDL- and LDL-cholesterol levels. There is no evidence that acute bouts of uninterrupted sedentary behaviour provide any positive changes in markers of cardiometabolic risk. However, the majority of studies identified by this paper focused on healthy young men, and it is therefore unclear whether these results will generalize to females or to other age groups. These findings suggest that uninterrupted bouts of sedentary behaviour should be avoided in order to prevent transient increases in metabolic risk. 

## Figures and Tables

**Algorithm 1:**
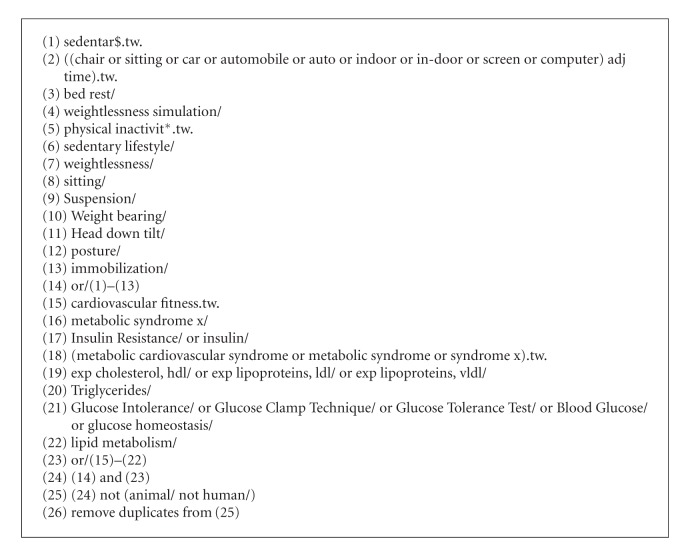
Medline search strategy.

**Table 1:** Characteristics of included studies.

Reference	Design	First author	Year	Country	*n* (M/F)	Age range	Mean age	Modality	Duration	Outcomes	Downs and Black
[37]	RCT	Yaroshenko	1998	Greece	30 (30/0)	22–26	24.8	BR	7 days	TG	24
[33]	RCT	Zorbas	1999	Bulgaria	30 (30/0)	22–26	24.3	BR	7 days	FG	24
[22]	RCO	Duran-Valdez	2008	USA	10 (2/8)	24–72	46.2	BR	2 days	FI, FG, IS	21
[11]	RCO	Stephens	2010	USA	12 (6/6)	19–32	26.1	SIT	1 day	FI, FG, IS	24
[39]	RCO	Dunstan	2012	Australia	19 (11/8)	45–65	53.8	SIT	5 hours	IS, GT	27
[42]	RCO	Nygaard	2009	Norway	13 (0/13)	>50		SIT	2 hours	GT	23
[21]	NT	Alibegovic	2010	Denmark	38 (38/0)		25.0	BR	7 days	FI	23
[31]	NT	Alibegovic	2009	Denmark	33 (33/0)		25.6	BR	7 days	FI	23
[23, 47]	NT	Blanc	2000	France	16 (8/8)		30.2	HDBR	6 days	FI, FG, IS, GT	23
[35]	NT	Dolkas	1977	USA	7 (7/0)	19–22	20.0	BR	4 days	FG	21
[25]	NT	Barbe	1999	France	8 (8/0)	23–31	27.1	HDBR	5 days	FI, FG	21
[10]	NT	Hamburg	2007	USA	20 (14/6)		30.7	BR	5 days	FI, FG, IS, TG	23
[20]	NT	Katkov	1979	Russia	5 (5/0)		34.0	BR	5 days	FG	17
[41]	NT	Kiilerich	2011	Denmark	6 (6/0)	22–36	28.7	BR	7 days	IS, GT	
[36]	NT	Ksinantova	2002	Slovakia	15 (15/0)		34.0	HDBR	4 days	FG	20
[29]	NT	Lipman	1972	USA	7 (7/0)	18–20		BR	3 days	FI, IS, GT	18
[32]	NT	Kanikowska	2010	Japan	8 (8/0)		27.0	HDBR	5 days	FI, FG, IS	
[27, 40]	NT	Mikines	1989	Denmark	6 (6/0)		25.0	BR	7 days	FI, FG, IS, GT	20
[30]	NT	Moro	2007	France	8 (8/0)	22–27	23.0	HDBR, SIT	4 hours	FI, FG	20
[34]	NT	Navasiolava	2010	Russia	8 (8/0)		23.0	BR	7 days	FG, TG, HDL, LDL	22
[26]	NT	Nygren	1997	Sweden	6 (6/0)		24.1	BR	1 day	FI, FG, IS	22
[19]	NT	Richter	1989	Denmark	5 (5/0)	22–24		CAST	7 days	IS	20
[24, 48, 49]	NT	Smorawinski	1996	Poland	29 (29/0)		20.10	BR	3 days	IS, GT	19
[28]	NT	Stuart	1988	USA	6 (6/0)	21–28	23.0	BR	7 days	FI, FG, IS, GT	22
[38]	NT	Yanagibori	1997	Japan	23 (13/10)	19–25		BR	3 days	IS, GT, TG, HDL, LDL	22

RCT: randomized controlled trial; RCO: randomized crossover; NT: nonrandomized trial; M: male; F: female; HDBR: head-down bed rest; 22BR: horizontal or head-up bed rest; SIT: sitting; CAST: casting; FG: fasting glucose; FI: fasting insulin; TG: triglycerides; HDL: HDL-cholesterol; LDL: LDL-cholesterol; IS: insulin sensitivity; GT: glucose tolerance.

When an intervention was described in more than one paper, the author name and year are taken from the earliest publication.

**Table 2:** Summary of key evidence.

Risk factor	Number of studies	Number of participants (M/F)	Size of effect	Quality of evidence
Insulin sensitivity	11	161 (118/43)	Moderate-to-Large	Moderate quality
Triglycerides	4	81 (65/16)	Moderate-to-Large	Moderate quality
Glucose tolerance	6	119 (83/36)	Moderate-to-Large	Moderate quality
HDL-cholesterol	3	51 (35/16)	Moderate	Very low quality
Fasting insulin	14	207 (187/20)	Small-to-Moderate	Very low quality
Fasting glucose	17	191 (163/28)	Small-to-Moderate	Very low quality
LDL-cholesterol	2	28 (22/6)	Moderate	Very low quality

## Abbreviations

HDL-cholesterol: High-density lipoprotein cholesterol
LDL-cholesterol: Low-density lipoprotein cholesterol
MET: Metabolic equivalent
HOMA: Homeostasis model of assessment
QUICKI: Quantitative insulin sensitivity check index
AUC: Area-under-the-curve.

## References

[1] Hu FB, Li TY, Colditz GA, Willett WC, Manson JE (2003). Television watching and other sedentary behaviors in relation to risk of obesity and type 2 diabetes mellitus in women. *Journal of the American Medical Association*.

[2] Rey-López JP, Vicente-Rodríguez G, Biosca M, Moreno LA (2008). Sedentary behaviour and obesity development in children and adolescents. *Nutrition, Metabolism and Cardiovascular Diseases*.

[3] Proper KI, Singh AS, Van Mechelen W, Chinapaw MJM (2011). Sedentary behaviors and health outcomes among adults: a systematic review of prospective studies. *American Journal of Preventive Medicine*.

[4] Katzmarzyk PT, Church TS, Craig CL, Bouchard C (2009). Sitting time and mortality from all causes, cardiovascular disease, and cancer. *Medicine and Science in Sports and Exercise*.

[5] Stamatakis E, Hamer M, Dunstan DW (2011). Screen-based entertainment time, all-cause mortality, and cardiovascular events: population-based study with ongoing mortality and hospital events follow-up. *Journal of the American College of Cardiology*.

[6] Warren TY, Barry V, Hooker SP, Sui X, Church TS, Blair SN (2010). Sedentary behaviors increase risk of cardiovascular disease mortality in men. *Medicine and Science in Sports and Exercise*.

[7] Lynch BM (2010). Sedentary behavior and cancer: a systematic review of the literature and proposed biological mechanisms. *Cancer Epidemiology Biomarkers and Prevention*.

[8] Hamilton MT, Hamilton DG, Zderic TW (2007). Role of low energy expenditure and sitting in obesity, metabolic syndrome, type 2 diabetes, and cardiovascular disease. *Diabetes*.

[9] Tremblay MS, Colley RC, Saunders TJ, Healy GN, Owen N (2010). Physiological and health implications of a sedentary lifestyle. *Applied Physiology, Nutrition and Metabolism*.

[10] Hamburg NM, McMackin CJ, Huang AL (2007). Physical inactivity rapidly induces insulin resistance and microvascular dysfunction in healthy volunteers. *Arteriosclerosis, Thrombosis, and Vascular Biology*.

[11] Stephens BR, Granados K, Zderic TW, Hamilton MT, Braun B (2011). Effects of 1 day of inactivity on insulin action in healthy men and women: interaction with energy intake. *Metabolism*.

[12] Bey L, Hamilton MT (2003). Suppression of skeletal muscle lipoprotein lipase activity during physical inactivity: a molecular reason to maintain daily low-intensity activity. *Journal of Physiology*.

[13] Wilkes JJ, Bonen A (2000). Reduced insulin-stimulated glucose transport in denervated muscle is associated with impaired Akt-*α* activation. *American Journal of Physiology*.

[14] Coderre L, Monfar MM, Chen KS (1992). Alteration in the expression of GLUT-1 and GLUT-4 protein and messenger RNA levels in denervated rat muscles. *Endocrinology*.

[15] Hamilton MT, Healy GN, Dunstan DW (2008). Too little exercise and too much sitting: inactivity physiology and the need for new recommendations on sedentary behavior. *Current Cardiovascular Risk Reports*.

[16] Bergouignan A, Rudwill F, Simon C, Blanc S ( 2011). Physical inactivity as the culprit of metabolic inflexibility: evidence from bed-rest studies. *Journal of Applied Physiology*.

[17] Downs SH, Black N (1998). The feasibility of creating a checklist for the assessment of the methodological quality both of randomised and non-randomised studies of health care interventions. *Journal of Epidemiology and Community Health*.

[18] Guyatt GH, Oxman AD, Vist GE, Kunz R, Falck-Ytter Y, Schünemann HJ (2008). GRADE: what is "quality of evidence" and why is it important to clinicians?. *British Medical Journal*.

[19] Richter EA, Kiens B, Mizuno M, Strange S (1989). Insulin action in human thighs after one-legged immobilization. *Journal of Applied Physiology*.

[20] Katkov VE, Chestukhin VV, Shefter LI (1979). Short-term immobilization of healthy men: right ventricular function and metabolism during graded exercise. *Cor et Vasa*.

[21] Alibegovic AC, Sonne MP, Højbjerre L (2010). The T-allele of TCF7L2 rs7903146 associates with a reduced compensation of insulin secretion for insulin resistance induced by 9 days of bed rest. *Diabetes*.

[22] Duran-Valdez E, De Serna DG, Schneider S, Amorim F, Burge M, Schade DS (2008). Metabolic effects of 2 days of strict bed rest. *Endocrine Practice*.

[23] Blanc S, Normand S, Pachiaudi C, Fortrat JO, Laville M, Gharib C (2000). Fuel homeostasis during physical inactivity induced by bed rest. *Journal of Clinical Endocrinology and Metabolism*.

[24] Smorawiński J, Kaciuba-Uściłko H, Nazar K (2000). Effects of three-day bed rest on metabolic, hormonal and circulatory responses to an oral glucose load in endurance or strength trained athletes and untrained subjects. *Journal of Physiology and Pharmacology*.

[25] Barbe P, Galitzky J, Thalamas C (1999). Increase in epinephrine-induced responsiveness during microgravity simulated by head-down bed rest in humans. *Journal of Applied Physiology*.

[26] Nygren J, Thorell A, Efendic S, Nair KS, Ljungqvist O (1997). Site of insulin resistance after surgery: the contribution of hypocaloric nutrition and bed rest. *Clinical Science*.

[27] Mikines KJ, Dela F, Tronier B, Galbo H (1989). Effect of 7 days of bed rest on dose-response relation between plasma glucose and insulin secretion. *American Journal of Physiology*.

[28] Stuart CA, Shangraw RE, Prince MJ, Peters EJ, Wolfe RR (1988). Bed-rest-induced insulin resistance occurs primarily in muscle. *Metabolism*.

[29] Lipman RL, Raskin P, Love T, Triebwasser J, Lecocq FR, Schnure JJ (1972). Glucose intolerance during decreased physical activity in man. *Diabetes*.

[30] Moro C, Pillard F, De Glisezinski I (2007). Atrial natriuretic peptide contribution to lipid mobilization and utilization during head-down bed rest in humans. *American Journal of Physiology*.

[31] Alibegovic AC, Højbjerre L, Sonne MP (2009). Impact of 9 days of bed rest on hepatic and peripheral insulin action, insulin secretion, and whole-body lipolysis in healthy young male offspring of patients with type 2 diabetes. *Diabetes*.

[32] Kanikowska D, Sato M, Iwase S (2010). Leptin and ghrelin levels in humans during physical inactivity induced by head-down bed rest. *Aviation Space and Environmental Medicine*.

[33] Zorbas YG, Yarullin VL, Denogradov SD, Afonin VB (1999). Plasma volume and biochemical changes in athletes during bed rest chronic hyperhydration. *Acta Astronautica*.

[34] Navasiolava NM, Dignat-George F, Sabatier F (2010). Enforced physical inactivity increases endothelial microparticle levels in healthy volunteers. *American Journal of Physiology*.

[35] Dolkas CB, Greenleaf JE (1977). Insulin and glucose responses during bed rest with isotonic and isometric exercise. *Journal of Applied Physiology Respiratory Environmental and Exercise Physiology*.

[36] Ksinantova L, Koska J, Kvetnansky R, Marko M, Hamar D, Vigas M (2002). Effect of simulated microgravity on endocrine response to insulin-induced hypoglycemia in physically fit men. *Hormone and Metabolic Research*.

[37] Yaroshenko YY, Zorban YG, Kuznetsov NK, Kakurin AG, Popov VK, Yazulin VL (1998). Changes in thyroid hormones and lipids in endurance trained volunteers during acute and rigorous bed rest conditions. *Wiener Klinische Wochenschrift*.

[38] Yanagibori R, Suzuki Y, Kawakubo K (1997). The effects of 20 days bed rest on serum lipids and lipoprotein concentrations in healthy young subjects. *Journal of Gravitational Physiology*.

[39] Dunstan D, Kingwell BA, Larsen R (2012). Breaking up prolonged sitting reduces postprandial glucose and insulin responses. *Diabetes Care*.

[40] Mikines KJ, Richter EA, Dela F, Galbo H (1991). Seven days of bed rest decrease insulin action on glucose uptake in leg and whole body. *Journal of Applied Physiology*.

[41] Kiilerich K, Ringholm S, Bienso RS (2011). Exercise-induced pyruvate dehydrogenase activation is not affected by 7 days of bed rest. *Journal of Applied Physiology*.

[42] Nygaard H, Tomten SE, Høstmark AT (2009). Slow postmeal walking reduces postprandial glycemia in middle-aged women. *Applied Physiology, Nutrition and Metabolism*.

[43] Colley RC, Garriguet D, Janssen I, Craig CL, Clarke J, Tremblay MS (2011). Physical activity of Canadian adults: accelerometer results from the 2007 to 2009 Canadian Health Measures Survey. *Health Reports*.

[44] Colley RC, Garriguet D, Janssen I, Craig CL, Clarke J, Tremblay MS (2011). Physical activity of Canadian children and youth: accelerometer results from the 2007 to 2009 Canadian Health Measures Survey. *Health Reports*.

[45] Carson V, Janssen I (2011). Volume, patterns, and types of sedentary behavior and cardio-metabolic health in children and adolescents: a cross-sectional study. *BMC Public Health*.

[46] Matthews CE, Chen KY, Freedson PS (2008). Amount of time spent in sedentary behaviors in the United States, 2003-2004. *American Journal of Epidemiology*.

[47] Blanc S, Normand S, Pachiaudi C, Duvareille M, Gharib C (2000). Leptin responses to physical inactivity induced by simulated weightlessness. *American Journal of Physiology*.

[48] Smorawinski J, Kaciuba-Uscilko H, Nazar K, Kaminska E, Korszun P, Greenleaf JE (1998). Comparison of changes in glucose tolerance and insulin secretion induced by three-day bed rest in sedentary subjects and endurance or strength trained athletes. *Journal of Gravitational Physiology*.

[49] Smorawinski J, Kubala P, Kaciuba-Uociako H, Nazar K, Titow-Stupnicka E, Greenleaf JE (1996). Effects of three day bed-rest on circulatory, metabolic and hormonal responses to oral glucose load in endurance trained athletes and untrained subjects. *Journal of Gravitational Physiology*.

